# *Staphylococcus epidermidis* alters macrophage polarization and phagocytic uptake by extracellular DNA release in vitro

**DOI:** 10.1038/s41522-024-00604-7

**Published:** 2024-11-20

**Authors:** Samira Weißelberg, Anna Both, Antonio Virgilio Failla, Jiabin Huang, Stefan Linder, Denise Ohnezeit, Patricia Bartsch, Martin Aepfelbacher, Holger Rohde

**Affiliations:** 1 Institut für Medizinische Mikrobiologie, Virologie und Hygiene, Universitätsklinikum Hamburg-Eppendorf (UKE), Hamburg, Germany; 2 UKE Microscopy Imaging Facility (Umif), Universitätsklinikum Hamburg-Eppendorf (UKE), Hamburg, Germany

**Keywords:** Biofilms, Cellular microbiology, Clinical microbiology, Pathogens

## Abstract

Biofilm formation shields *Staphylococcus epidermidis* from host defense mechanisms, contributing to chronic implant infections. Using wild-type *S. epidermidis* 1457, a PIA-negative mutant (1457-M10), and an eDNA-negative mutant (1457Δ*atlE*), this study examined the influence of biofilm matrix components on human monocyte-derived macrophage (hMDM) interactions. The wild-type strain was resistant to phagocytosis and induced an anti-inflammatory response in hMDMs, while both mutants were more susceptible to phagocytosis and triggered a pro-inflammatory response. Removing eDNA from the 1457 biofilm matrix increased hMDM uptake and a pro-inflammatory reaction, whereas adding eDNA to the 1457Δ*atlE* mutant reduced phagocytosis and promoted an anti-inflammatory response. Inhibiting TLR9 enhanced bacterial uptake and induced a pro-inflammatory response in hMDMs exposed to wild-type *S. epidermidis*. This study highlights the critical role of eDNA in immune evasion and the central role of TLR9 in modulating macrophage responses, advancing the understanding of implant infections.

## Introduction

Over the past decades, the ubiquitous human skin colonizing bacterium *Staphylococcus epidermidis* has been documented as a major cause of hospital-acquired infections. Interestingly, as compared to its close relative *Staphylococcus aureus*, *S. epidermidis* lacks dedicated factors facilitating aggressive invasion^1,2^. In fact, a prerequisite for *S. epidermidis* infections to occur are predisposing host conditions, e.g., preterm birth and severe immunosuppression. The most important predisposing condition, however, is implantation of a medical device (i.e., prosthetic joints, central venous catheters, breast implants, prosthetic heart valves). Over the past three decades, *S. epidermidis* has emerged as the leading causative pathogen in central line associated blood stream infections (CLABSI) and prosthetic joint infections (PJI), causing ~30% of these infections^3–7^.

Successful invasion and persistence within the host is critically dependent on the ability of the pathogen to evade clearance by innate or adaptive immune effector mechanisms. Strikingly, *S. epidermidis* effectively lacks immune evasion mechanisms that have been identified in *S. aureus*^8–10^. Therefore, outside its natural skin niche environment *S. epidermidis* usually is readily cleared by host innate immunity^11^. Thus, the success of *S. epidermidis* to persist in the hostile host environment during foreign material-associated infections must be explained by the unique pathogenesis of implant-associated infections. The ability of *S. epidermidis* to survive in the hostile host environment is solely dependent on its capacity to form three-dimensional biofilms on abiotic surfaces^12^. This link has recently been underlined by genomic studies showing that genes related to biofilm formation are enriched in *S. epidermidis* clones ST2, ST5, and ST23, which are the most frequently isolated sequence types^13,14^. The concept that biofilm formation is an essential factor for intra-host persistence has therefore stimulated great interest in the molecular mechanisms underlying *S. epidermidis* biofilm formation and their specific role in the interaction with host immune cells.

From the detailed analysis of *S. epidermidis* multicellularity, the complex picture of a multi-stage developmental process of biofilm formation evolved, characterized by the functional activities of distinct bacterial factors^15,16^. A significant functional hallmark of biofilm formation is production and assembly of an extracellular matrix, consisting of polysaccharides (e.g., polysaccharide intercellular adhesion [PIA]), proteins (e.g., accumulation associated protein [Aap]), and extracellular DNA (eDNA)^17–21^. The extracellular matrix is of key importance during biofilm formation, as the matrix components provide a mechanical scaffold that tightly integrates and embeds single *S. epidermidis* cells into the multicellular community^22,23^.

Biofilm formation in general has a profound impact onto staphylococcal interactions with professional phagocytes, e.g., macrophages^24,25^. Normally, in acute infections caused by rapidly dividing planktonic staphylococci, macrophages are able to efficiently recognize and ingest staphylococci, ultimately leading to the killing and clearance of the invading pathogen.^26^. In addition, macrophages use cell surface receptors TLR2 and CD14 to efficiently recognize staphylococcal cell wall components (e.g., peptidoglycan, lipoproteins, lipoteichoic acid [LTA]), resulting in a specific transcriptional response in which NF-kB drives the expression of inflammatory genes, including pro-inflammatory cytokines such as TNFα, IL-6, and IL-1β^26^. Consequently, this pro-inflammatory macrophage activation not only enhances the macrophage’s ability to kill staphylococcal cells but also, by attracting polymorphonuclear neutrophils, creates a general antibacterial milieu that limits the spread of pathogens at the site of invasion^27^.

In contrast to findings made with planktonic bacteria, evidence indicates a selective failure of macrophages to eradicate surface-organized *Staphylococcus spp*. in general and *S. epidermidis* in specific, linking a multicellular biofilm phenotype with immune evasion^28,29^. Several concepts on the molecular mechanisms contributing to biofilm-dependent staphylococcal immune evasion have been brought into play. The size of bacterial aggregation itself supports protection against phagocytosis^30,31^, and the inability of professional phagocytes to break down the biofilm through expression of matrix-degrading enzymes may contribute to the process referred to as “frustrated phagocytosis”^32^. Moreover, it has been reported that PIA protects *S. epidermidis* from antimicrobial peptides, either by repulsion of antimicrobial peptides, or by sequestering anionic peptides^33^. PIA also contributes to the protection of *S. epidermidis* from complement mediated killing, activated via the classical or alternative pathway^34,35^. Work from our group using the mouse macrophage-like cell line J774A.1 showed that irrespective of the absence or presence of PIA, also Aap and Embp-dependent *S. epidermidis* biofilms induced less NF-kB activation and *IL1B* expression compared to isogenic biofilm-negative mutants. Importantly, not only did biofilm-forming *S. epidermidis* elicit a weaker pro-inflammatory response in macrophages, but prior exposure to *S. epidermidis* biofilms resulted in cross-desensitization of macrophages to stimulation with the TLR-4 activator LPS^36^. These findings are in line with the idea that in addition to providing a physical barrier for innate immune effector mechanisms, staphylococcal biofilms generally impact the innate immune activation by skewing pro-inflammatory bactericidal responses towards anti-inflammatory host reactions, thereby supporting bacterial persistence^37–39^. In *S. aureus*, anti-inflammatory macrophage priming has been partially linked to metabolic host-pathogen crosstalk, where lactate released from the biofilm is actively transported into the macrophage to inhibit histone deacetylase 11 (HDAC11)^40^. This ultimately results in IL10 production which is important for polarizing the anti-inflammatory biofilm milieu^41^. At present, however, the molecular mechanisms underlying *S. epidermidis* escape from phagocytosis and anti-inflammatory macrophage priming remain enigmatic.

This study tested the hypothesis that the specific biofilm matrix composition is critical for the interaction between *S. epidermidis* and macrophages. Using biofilm-forming wild-type *S. epidermidis* 1457 producing PIA and eDNA, and mutants deficient in PIA or eDNA, the importance of specific matrix components for biofilm architecture was verified and the consequences for phagocytic uptake and macrophage polarization were analyzed. Evidence is presented that in *S. epidermidis*, it is not biofilm formation and cell aggregation per se that is ultimately responsible for the failure of *S. epidermidis* uptake and anti-inflammatory macrophage polarization. Instead, the presence of eDNA within a biofilm matrix scaffold, coupled with TLR9 activation, is essential for promoting biofilm-associated interference with phagocytosis and the induction of an anti-inflammatory macrophage phenotype. These findings significantly extend the current understanding of *S.* epidermidis–macrophage interactions^42^.

## Results

### Matrix components shape *S. epidermidis* biofilm architecture

**Fig. 1:**
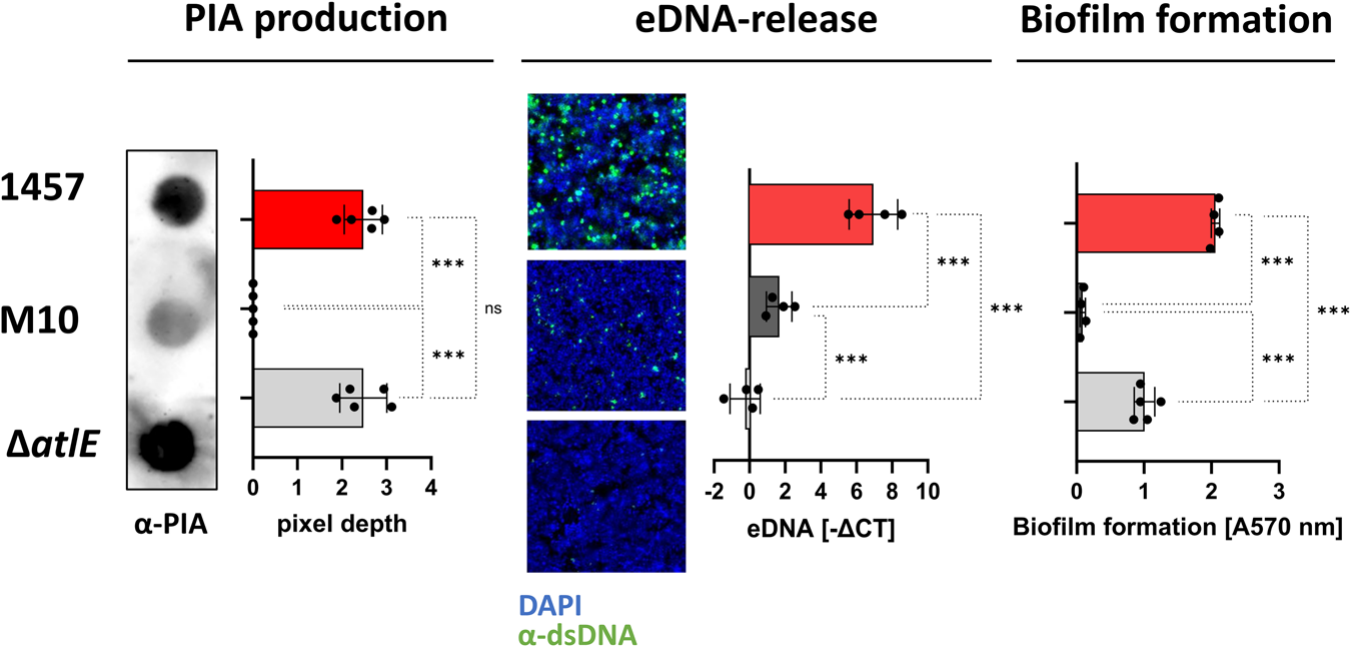
Presence of PIA and eDNA in *S. epidermidis* biofilms. **(PIA production)** Dot blot analysis of cell surface-associated PIA. Surface-bound PIA was extracted from sessile cultures, and spotted onto PVDF membranes. PIA was detected using a polyclonal rabbit α-PIA antiserum and HRP-conjugated anti-rabbit IgG. Columns represent mean pixel depth values for *S. epidermidis* 1457 and 1457Δ*atlE* after subtraction of 1457-M10 results. Pixel depth was calculated as a proxy for PIA quantity using the ImageQuantTL software package. Data are representative of five biological replicates. **(eDNA release)** Left: eDNA detection in sessile *S. epidermidis* cultures after 24 h static growth using immunofluorescence staining. eDNA was stained with a rabbit α-dsDNA antibody, a conjugated α-rabbit A488 secondary antibody, bacterial cells were stained using DAPI. Scale bar: 5 µm. Right: qRT-PCR-based quantification of eDNA from sessile cultures of *S. epidermidis* 1457, 1457-M10, and 1457Δ*atlE*. eDNA was extracted from sessile overnight cultures grown in TSB and preparations were subjected to qRT-PCR analysis using *gyrB-*specific primers. **(Biofilm formation)** *S. epidermidis* 1457, 1457-M10, and 1457Δ*atlE* were tested for biofilm formation and cell adherence using the microtiter plate assay. Columns represent mean absorption at 570 nm from four biological replicates; error bars depict standard deviation. Results are presented as mean -ΔCt values from four biological replicates. Pairwise comparison was performed using one-way ANOVA. ns: not significant, *P ≤ 0.05; **P ≤ 0.01; ***P ≤ 0.001.

*S. epidermidis* biofilm formation essentially depends on the production of a biofilm matrix to support bacterial cell aggregation, with polysaccharide PIA and extracellular DNA (eDNA) being important matrix components. Using wild-type *S. epidermidis* 1457 and corresponding mutants defective in PIA production (1457-M10) or eDNA release (1457Δ*atlE*) (Table 1), the importance of these factors for biofilm formation and structuring of sessile cell consortia was tested (Fig. 1). Under these conditions, *S. epidermidis* 1457 and 1457Δ*atlE*, but not 1457-M10 produced cell surface-associated PIA (Fig. 1). Immunofluorescence analysis using a dsDNA-specific antibody showed that sessile 1457Δ*atlE* cell consortia were completely devoid of detectable eDNA (Fig. 1). In sharp contrast, eDNA was abundant in sessile *S. epidermidis* 1457 cultures, whereas only small amounts of eDNA were detected in 1457-M10 compared to the wild type (Fig. 1). Findings from microscopic eDNA detection were corroborated by quantification of cell surface-associated eDNA in *S. epidermidis* using qPCR (Fig. 1), indicating that the release of eDNA is *atlE*-dependent, while production of a PIA-containing biofilm matrix is necessary for eDNA enrichment within a sessile *S. epidermidis* population. A microtiter plate assay, allowing for quantification of adherent cell consortia, showed that cells from wild-type *S. epidermidis* 1457 and mutant 1457Δ*atlE* remained adherent despite the application of shear stress, i.e., both strains assembled a bona fide surface-adherent biofilm (Fig. 1). In contrast, cells of PIA-negative mutant 1457-M10 were readily removed, resulting in a biofilm-negative phenotype (Fig. 1). Notably, the absence of eDNA in 1457Δ*atlE* was associated with reduced biofilm formation as compared to wild-type 1457 (Fig. 1).

**Table 1:** Strains and mutants used in this study

Strain	Relevant characteristic	Reference
*S. epidermidis* 1457^a^	PIA-producing, biofilm-positive, isolated from central venous catheter infection.	^87^
*S. epidermidis* 1457-M10^a^	Biofilm-negative mutant with Tn*917* in *icaA*, no PIA synthesis.	^97^
*S. epidermidis* 1457Δ*atlE*^a^	Deletion mutant in major autolysin encoding *atlE*, defect in DNA release.	^89^

^a^In some experiments, strains carrying pCM29 to allow for constitutive *gfp* expression, were employed^98^.

**Fig. 2:**
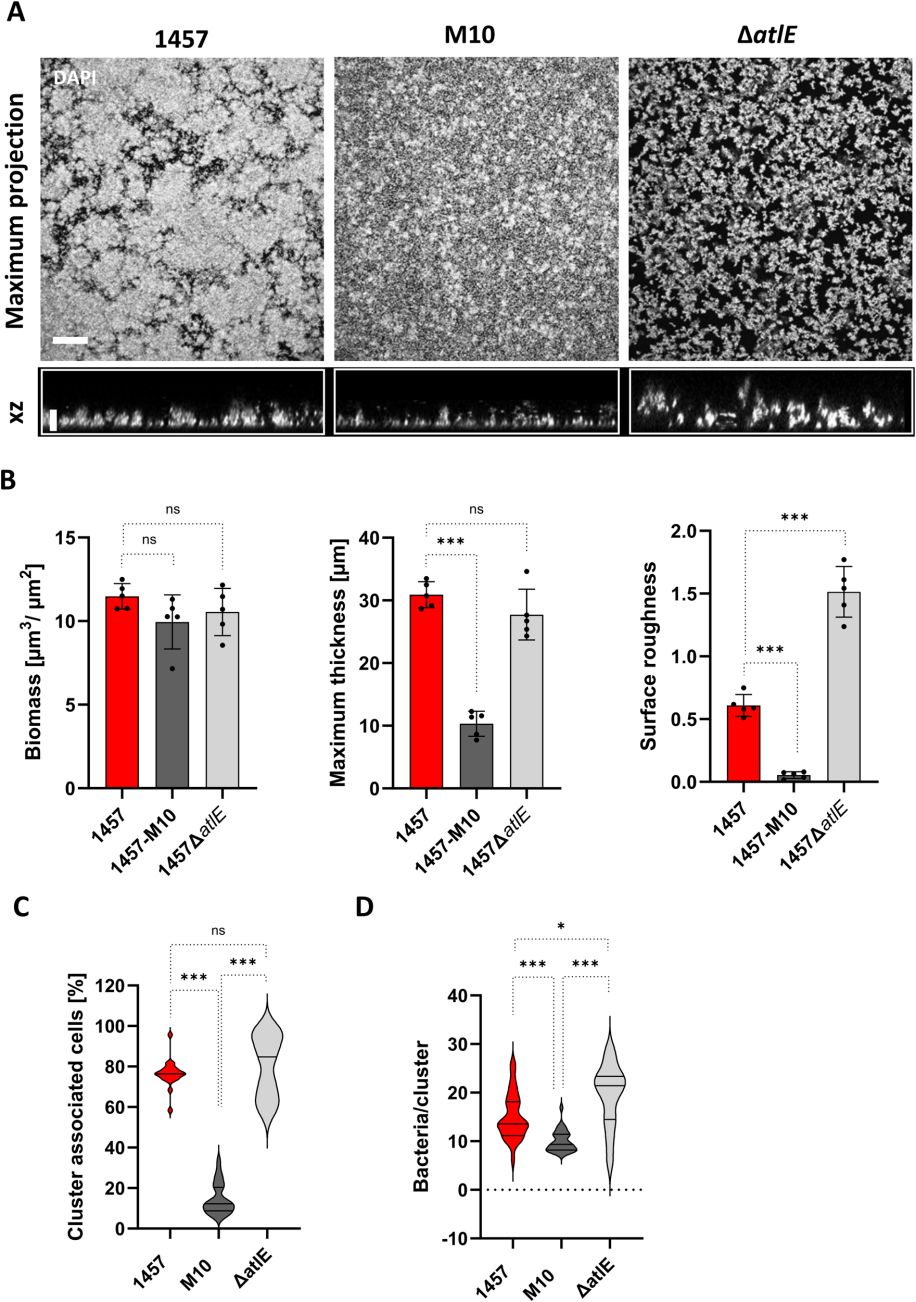
Structure of multicellular *S. epidermidis* assemblies. **A** CLSM analysis of sessile *S. epidermidis* cultures after overnight growth in TSB. Bacteria were grown for 24 h, stained with DAPI and subjected to microscopic imaging. The upper panel shows the maximum projection of DAPI channel and the lower panel shows the corresponding XZ-view (scale bar: 10 µm). **B** Biomass, maximum thickness and surface roughness of 1457, 1457-M10 and 1457Δ*atlE*. At least five confocal images of three independent 24 h sessile cultures per strain were processed using the Comstat add-on in ImageJ. Values represent mean with standard deviation. Pairwise comparison was assessed using one-way ANOVA. **C** Quantification of bacterial cells organized in cell clusters. Using Imaris and Matlab software packages the proportion of bacteria organized in cell clusters >5 cells was determined by analysis of at least 10 CLSM images per strain. In total, at least 30 images from three independent experiments were analyzed. Statistical analysis was performed using one-way ANOVA with Bonferroni´s correction for multiple testing. **D** Determination of bacterial aggregate sizes. The size of bacterial cell aggregates was determined using Imaris and Matlab software packages. Per strain, at least 30 images from three independent experiments were analyzed. Statistical analysis was performed using one-way ANOVA with Bonferroni´s correction for multiple testing. ns not significant, *P* > 0.05; *P ≤ 0.05; **P ≤ 0.01; ***P ≤ 0.001.

To gain insights into the spatial cell organization associated with sessile bacterial growth, static cultures of *S. epidermidis* 1457 and corresponding mutants 1457-M10 and 1457Δ*atlE* were analyzed by confocal laser scanning microscopy (CLSM). *S. epidermidis* 1457 assembled into a densely packed cell architecture, characterized by tower-like formations resulting in relevant surface roughness (Fig. 2A, B). PIA-producing, eDNA-negative 1457Δ*atlE* still formed surface adherent, densely packed cell aggregates with a maximum thickness comparable to that of wild-type 1457, however, 1457Δ*atlE* showed a significantly greater surface roughness (Fig. 2B). In contrast, sessile populations of *S. epidermidis* 1457-M10, while mounting a similar biomass compared to *S. epidermidis* 1457 and 1457Δ*atlE*, were characterized by an approximately tenfold smoother surface and approximately 3-fold lower maximum thickness compared to the wildtype (Fig. 2B). These findings are indicative that the lack of PIA-mediated, intercellular adhesive properties in 1457-M10 results in a compact, mostly unstructured accumulation of loosely attached bacterial cells. Indeed, analysis of aggregative behavior showed that, relative to the total number of bacteria, the percentage of cells organized in aggregates (defined clusters as containing five or more cells) was significantly higher in *S. epidermidis* 1457 (76.44% ± 6.77) and 1457Δ*atlE* (80.17% ± 15.42) compared to 1457-M10 (14.6% ± 7.65) (Fig. 2C). The mean cluster size in mutant 1457-M10 (9.98 ± 2.18 cells/cluster) was significantly (*P* < 0.0001) smaller compared to *S. epidermidis* 1457 and 1457Δ*atlE*, while clusters of the latter were significantly larger compared to the 1457 (19.45 ± 6.50 vs. 15.08 ± 4.71 cells/cluster, *P* < 0.017) (Fig. 2D). In summary, these results provide strong evidence that both, wild-type *S. epidermidis* 1457 and mutant 1457Δ*atlE* assemble cell populations that formally represent bona fide surface-adherent biofilms. By contrast, sessile 1457-M10 cultures represent unstructured, loosely associated piles of sedimented cells. Altered biofilm morphology and cell clustering characteristics indicate that eDNA, while not essential for cell aggregation and biofilm accumulation, significantly modulates intercellular adhesion properties and structuring of the biofilm architecture.

### Macrophage uptake of *S. epidermidis* is impacted by biofilm matrix composition

**Fig. 3:**
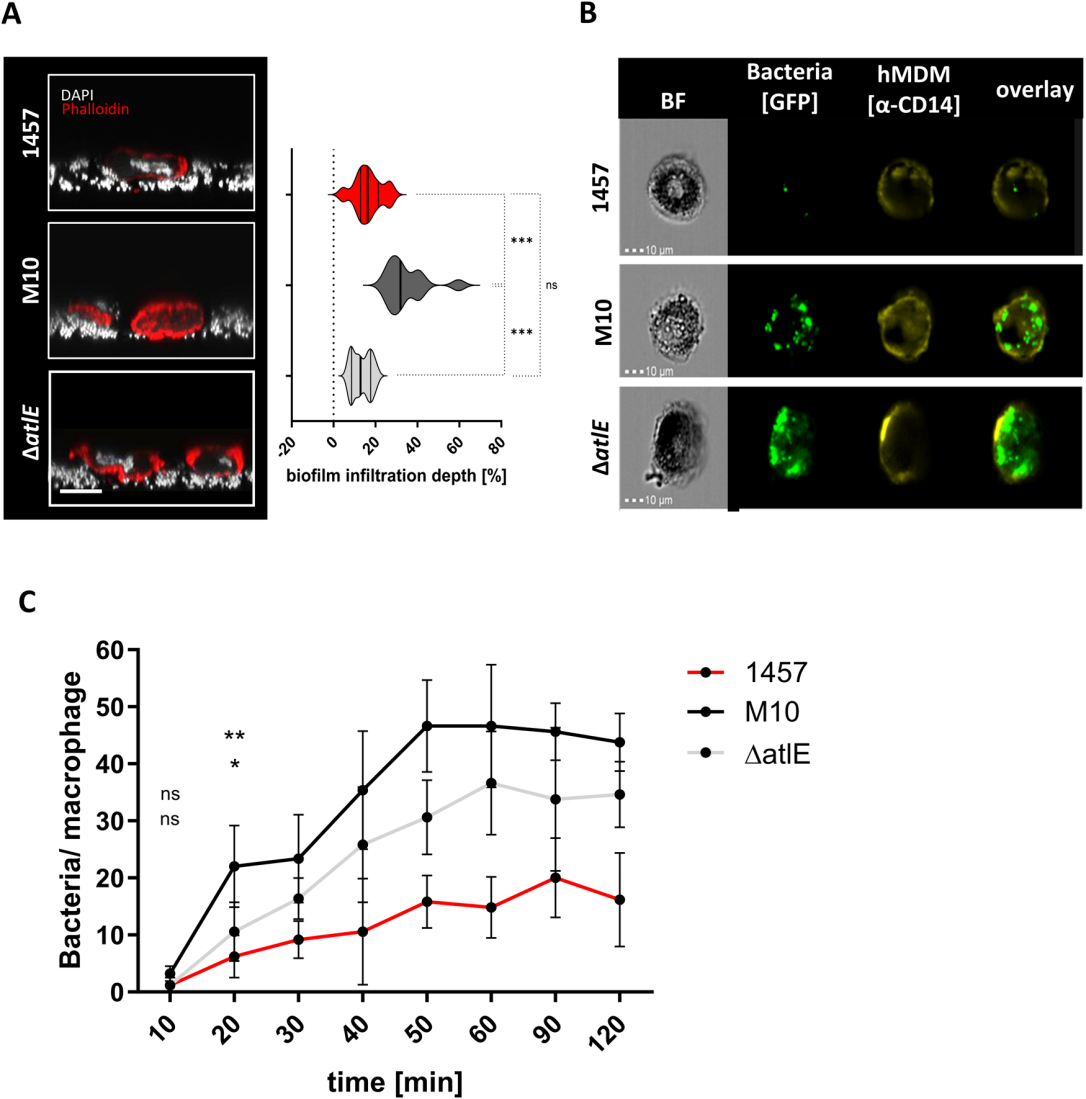
Phagocytosis of *S. epidermidis.* **A** Imaging of macrophage–S*. epidermidis* interactions: hMDM were infected with sessile *S. epidermidis* 1457, 1457-M10 and 1457∆*atlE* cultures, and the macrophage- bacteria interface was analyzed 2 h after infection using CLSM. Left panel: Representative image of macrophages interacting with sessile *S. epidermidis* 1457, 1457-M10, and 1457Δ*atlE* cultures. Co-cultures were stained using DAPI (white) and Phalloidin (red). Scale: 10 µm. Right panel: Quantitative analysis of macrophage infiltration depth. Based on three experiments using macrophages from independent donors, the mean depth of macrophage biofilm infiltration relative to the mean height of the respective sessile cultures was estimated using BiofilmQ and the Comstat plug-in in ImageJ. At least 10 images per experimental condition were analyzed. **B** Image Stream analysis of *S. epidermidis* uptake into hMDM. hMDM were infected with static cultures of GFP-expressing *S. epidermidis* 1457, 1457-M10, and 1457Δ*atlE* at a MOI 50. After 20 min infection was stopped, macrophages were stained with α-CD14 IgG coupled to A514, and cells were analyzed on an Imaging Flow Cytometer. Representative pictures of three biological replicates are shown. **C** Time-dependent uptake of *S. epidermidis* into hMDM. hMDM were infected (MOI 50) with sessile cultures of GFP-expressing 1457, 1457-M10, and 1457Δ*atlE*. After indicated time points, infection was stopped and extracellular bacteria were discriminated from engulfed bacteria by outside staining using a rabbit α-*Staphylococcus epidermidis* antiserum and an α-rabbit IgG coupled to AF568. Bacterial uptake by macrophages was evaluated using CLSM and number of intracellular bacteria was determined. At least five pictures (minimum 5 macrophages) per strain and time point were analyzed. Pairwise comparison with wild-type strain 1457 was carried out using one-way ANOVA. Upper significance: 1457 vs. 1457-M10, lower significance: 1457 vs. 1457Δ*atlE*. ns not significant, *P* > 0.05; *P ≤ 0.05; **P ≤ 0.01; ***P ≤ 0.001.

Macrophages belong to the first line of immune defense during bacterial infections, but apparently fail to control *S. epidermidis* in foreign material-associated infections^26,43^. Based on the finding that 1457, 1457-M10, and 1457Δ*atlE* differ specifically in their clustering ability related to the differential production of biofilm matrix components, a series of experiments was designed to study the importance of PIA and eDNA on *S. epidermidis* interactions with human monocyte-derived macrophages (hMDM). To this end, an in vitro infection model was employed in which sessile *S. epidermidis* are brought into direct contact with hMDM. CLSM imaging showed that after 2 h, hMDM were immersed within the cell architecture of wild-type *S. epidermidis* 1457 and 1457-M10 and 1457Δ*atlE*. The relative penetration depth (i.e., mean position of hMDM within the sessile culture relative to the mean height of the sessile population), however, was greater during hMDM interaction with 1457-M10 (mean penetration depth 34%) than with 1457 and 1457Δ*atlE* cultures (mean penetration depth 16.6% and 13.3%, respectively) (Fig. 3A). This indicates that macrophages can come into direct contact with sessile *S. epidermidis* cells and invade the bacterial communities, but that this ability is compromised by the production of an extracellular matrix. Analysis of macrophage viability found no evidence for potential cell toxicity exerted from any of the *S. epidermidis* strains employed (Supplementary Fig. 1).

Quantification of phagocytosis during the first 120 min after start of co-incubation provided evidence for a time-dependent increase in intracellular bacteria (Fig. 3B). However, the rate of internalization of *S. epidermidis* cells was strain-dependent: between 10 and 60 min, an average of 0.9 *S. epidermidis* 1457-M10 and 0.7 *S. epidermidis* 1457Δ*atlE* cells were internalized per minute, but only 0.3 *S. epidermidis* 1457 cells. Approximately after 50–60 min of infection, no substantial additional increase of internalized bacteria was found (Fig. 3B). At 120 min of infection, the mean number of intracellular bacteria for 1457-M10 and 1457Δ*atlE* were 43.8 ± 5.1 and 34.6 ± 5.7 [mean bacteria/hMDM], respectively (Fig. 3B, C), compared to a maximum of 16.2 ± 8.2 bacteria per macrophage in *S. epidermidis* 1457 (Fig. 3B, C). Given the aggregative, biofilm-forming properties of *S. epidermidis* 1457Δ*atlE*, these data collectively suggest that the macrophage phagocytosis efficiency of biofilm-forming *S. epidermidis* is predominantly modulated by the composition of the biofilm matrix.

### Composition of the biofilm matrix shapes the pro-inflammatory activation of macrophages

Based on the differential uptake of the *S. epidermidis* strains, it was hypothesized that it is not biofilm formation per se, but rather the composition of the biofilm matrix and the presence of eDNA that is critical in shaping the interaction between *S. epidermidis* and macrophages. To further support this hypothesis, experiments were performed to investigate the differential induction of pro- and anti-inflammatory hMDM phenotypes after infection with *S. epidermidis*^44^.

**Fig. 4:**
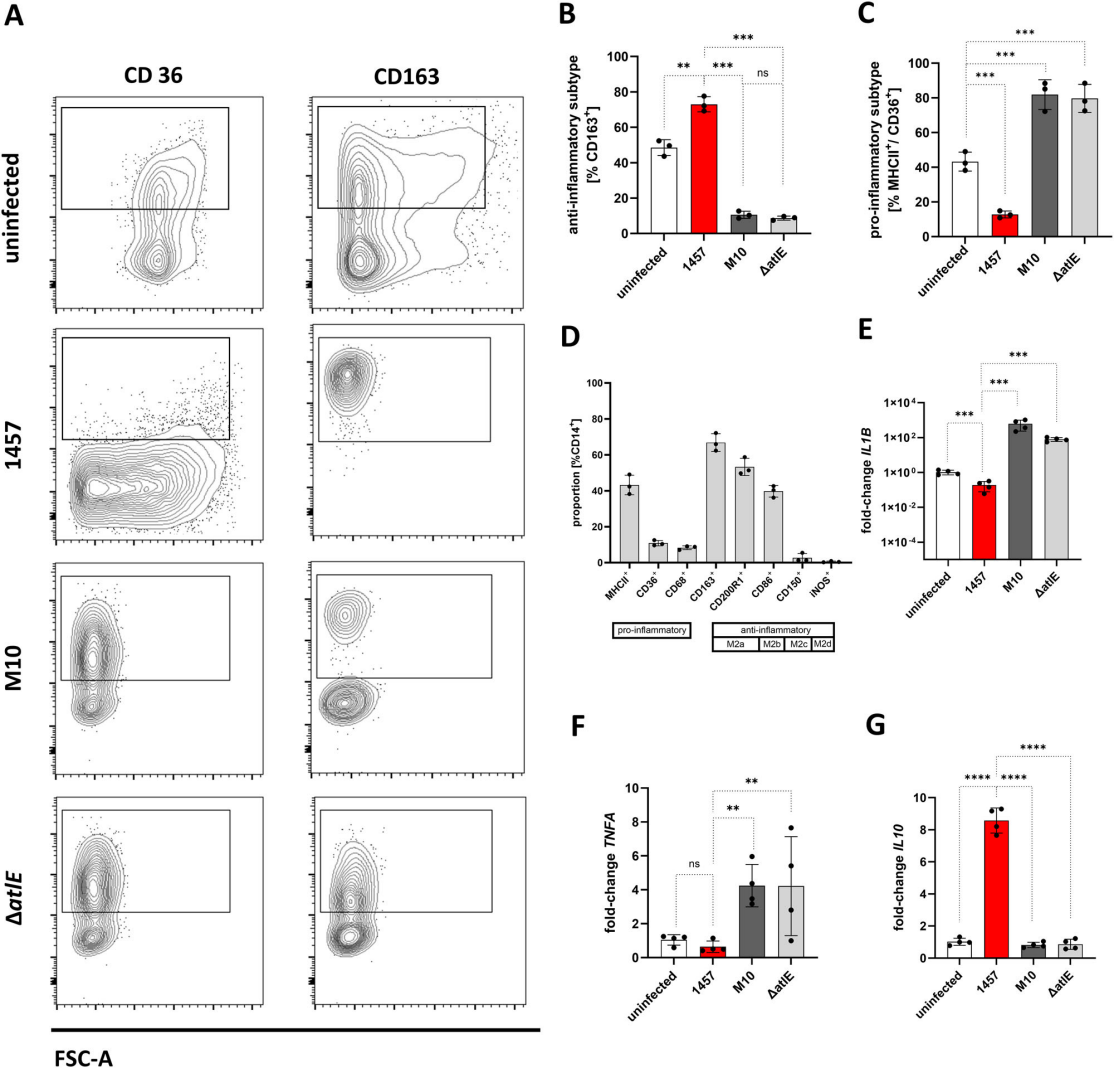
Macrophage polarization after infection with sessile *S. epidermidis* cultures. **A** Representative FACS plots and quantification of CD14^+^ positive primary human macrophages uninfected and after infection with *S. epidermidis* 1457, 1457-M10, and 1457Δ*atlE*. Cell surface markers were analyzed by immunocytostaining of CD14, CD68, CD36, MHCII and CD163. **B**, **C** Quantitative FACS analysis of pro- and anti-inflammatory subtype distribution after hMDM infection with *S. epidermidis* 1457, 1457-M10, and 1457Δ*atlE* (MOI 50). Columns represent mean proportion of CD163-positive and CD36^+^MHCII^+^ cells, respectively, in the total number of CD14^+^ cells. Error bars indicate standard deviation. Pairwise comparison was done using one-way ANOVA. **D** Detailed anti-inflammatory subtype analysis of hMDM after infection with *S. epidermidis* 1457 (MOI 50). Using immunocytostaining the percentage of hMDM cells positive for CD14, MHCII, CD36, CD68, CD163, CD200R1, CD86, CD150, iNOs was quantified using FACS. **E**–**G** Relative quantification of *IL1B*, *TNFA*, and *IL10* gene expression in hMDM after infection with *S. epidermidis* 1457, 1457-M10, and 1457Δ*atlE* using *GAPDH* as a reference housekeeping gene. Columns show mean fold changes determined by the comparative C(t) method of three independent donors relative to uninfected hMDM control. Bars represent standard deviation. Statistical analysis was performed using one-way ANOVA. ns not significant, *P* > 0.05; *P ≤ 0.05; **P ≤ 0.01; ***P ≤ 0.001.

To this end, flow cytometry analysis was employed to quantify CD36 (marker for pro-inflammatory polarization) or CD163 (a marker for anti-inflammatory polarization^25^ expressing hMDM (CD14^+^/MHCII^+^) populations after infection with *S. epidermidis* 1457, 1457-M10, and 1457Δ*atlE*. The un-stimulated hMDM control population consisted of almost equal pro- and anti-inflammatory proportions (CD14 + /CD36+ cells: 38.5% CD14 + /CD163+ cells: 48.54%) (Fig. 4A, B). After infection with *S. epidermidis* 1457, the proportion of CD163+ macrophages rose to 73 ± 4.2%, while the percentage of CD36+ cells declined to 12.79 ± 2.01% (Fig. 4A, B). In contrast, both, 1457-M10 and 1457Δ*atlE*, induced a predominant pro-inflammatory polarization, with 81.8 ± 8.5% and 79.7 ± 8.0% of hMDM being CD36 + , respectively (Fig. 4A, B). The percentage of CD163+ hMDM cells declined to 10.53 ± 2.02% (1457-M10) and 8.68 ± 1.11% (1457Δ*atlE*). Thus, eDNA is critical in shaping the hMDM activation during interactions with *S. epidermidis*, inducing an anti-inflammatory hMDM phenotype.

According to the expression of CD163, CD200R1, CD86, CD150, and iNOS, anti-inflammatory macrophages are further differentiated into four distinct sub-types^45^. After infection with *S. epidermidis* 1457, macrophages predominantly shifted to CD163^+^/CD200R1^+^ (CD163^+^: 66.84% ± 4.95. CD200R1^+^: 53.25% ± 4.799) and CD86^+^ (CD86^+^: 39.71% ± 3.23) populations, i.e., a phenotype referred to as M2a/M2b^45^ (Fig. 4D). CD150^+^ and iNOS^+^ populations were detected, however in significantly smaller proportions (CD150^+^: 2.69% ± 2.40; iNOS^+^: 0.50% ± 0.36).

To correlate differential induction of hMDM phenotypes as determined by surface marker expression analysis with mRNA expression of inflammatory and anti-inflammatory cytokines, induction of pro- (*TNFA*, *IL1B*) and anti-inflammatory (*IL10*) cytokine expression was quantified using qPCR (Fig. 4E–G). Compared to *S. epidermidis* 1457, hMDM infection with biofilm-negative mutant 1457-M10 as well as biofilm-positive, eDNA-negative 1457Δ*atlE* induced a significantly stronger expression of *TNFA*- and *IL1B*-response (Fig. 4E, F). In contrast, hMDM infection with *S. epidermidis* 1457 induced a significantly higher expression of *IL10* compared to *S. epidermidis* 1457-M10 and 1457Δ*atlE* (Fig. 4G).

### Presence of eDNA is essential for impaired *S. epidermidis* uptake and anti-inflammatory macrophage priming

**Fig. 5:**
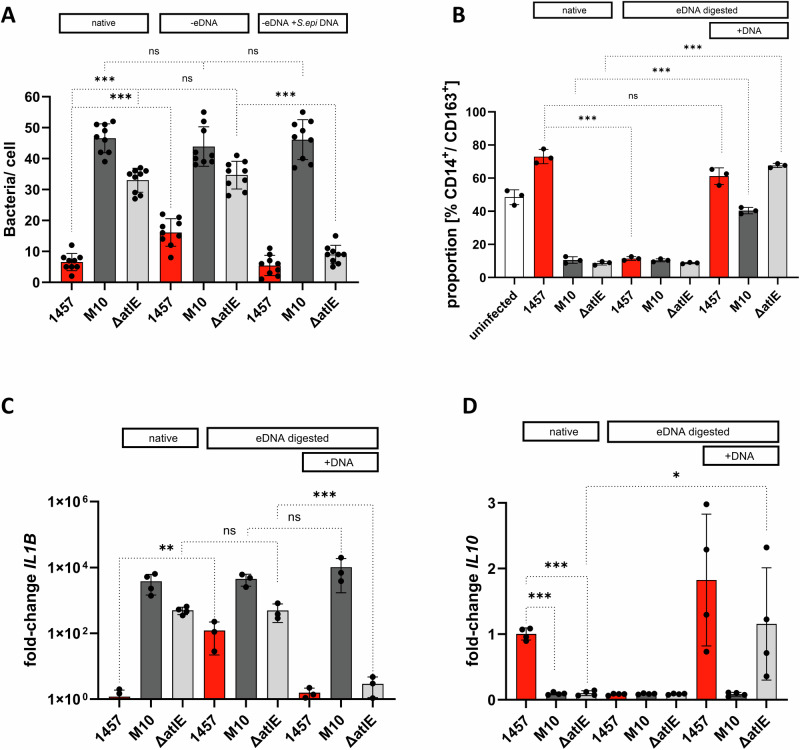
Uptake of *S. epidermidis* and inflammatory activation of hMDM after eDNA removal. **A** Uptake of native, DNaseI-treated and eDNA supplemented, sessile *S. epidermidis* by hMDM. Uptake of GFP-expressing *S. epidermidis* was quantified by CLSM after DAPI staining and specific detection of extracellular bacteria *S. epidermidis* using a rabbit α-*Staphylococcus epidermidis* antiserum and an α-rabbit IgG coupled to AF568. At least nine images per condition and donor (*n* = 3) were recorded. **B** Comparison of anti-inflammatory priming of hMDM after infection with sessile *S. epidermidis* cultures after DNaseI treatment, and eDNA supplementation. Untreated cultures served as a control. CD14^+^ and CD163^+^ cells were detected by immunocytostaining and were quantified using FACS. Columns represent the mean proportion of CD163^+^ cells within the population of CD14+ cells. Error bars indicate standard deviation. **C**, **D** Relative quantification of *IL1B*, and *IL10* expression in hMDM after infection with *S. epidermidis* 1457, 1457-M10, and 1457Δ*atlE* using *GAPDH* as a reference housekeeping gene. Cultures were untreated, treated with DNaseI, or DNaseI-treated and supplemented with chromosomal DNA. Columns represent mean fold changes relative to hMDM infected with *S. epidermidis* 1457, and based on the analysis of hMDM from four independent donors. Bars represent standard deviation. Statistical analysis was performed using one-way ANOVA with Bonferroni correction for multiple testing on ΔCt values. ns: not significant, *P* > 0.05; *P ≤ 0.05; **P ≤ 0.01; ***P ≤ 0.001.

Building on the results from phagocytic uptake and pro-inflammatory differentiation of hMDM, the hypothesis was followed that eDNA is significantly involved in modulating *S. epidermidis*–hMDM interactions, being decisive for the induction for an anti-inflammatory hMDM activation. To test this hypothesis infection experiments were carried out in which eDNA was enzymatically removed from sessile overnight *S. epidermidis* cultures by DNaseI treatment, and phagocytic uptake was assessed after infection with DNaseI-treated, sessile *S. epidermidis* cultures in comparison to untreated controls (Fig. 5A). Enzymatic removal of eDNA from 1457 biofilms (Supplementary Fig. 2) resulted in a significant increase of bacterial uptake (DNaseI-treated culture: 16.11 ± 4.45, native control culture: 6.55 ± 2.78 [mean bacteria/hMDM]) (Fig. 5A). In contrast, DNaseI treatment did not change the phagocytic uptake of 1457-M10 (DNaseI-treated: 19,88 ± 4.80; native control culture: 22.0 ± 5.78 [mean number of bacteria/hMDM]) and 1457Δ*atlE* (DNaseI-treated: 14.66 ± 4.47; native control culture: 11.89 ± 4.59 [mean number of bacteria/hMDM]) (Fig. 5A).

We further reasoned that, if eDNA indeed is of functional relevance to provide protection from phagocytosis, addition of exogenous chromosomal *S. epidermidis* DNA should rescue the phenotype of not only DNaseI-treated *S. epidermidis* 1457, but also mutant 1457Δ*atlE*. The addition of DNA and its integration into the matrix of to DNaseI-treated wild-type 1457 (Supplementary Fig. 2) partially restored phagocytosis resistance towards hMDM. Moreover, exogenous DNA also integrated into the biofilm matrix of 1457Δ*atlE* (Supplementary Fig. 2), and resulted in a level of phagocytosis resistance comparable to wild-type *S. epidermidis* 1457 (Fig. 5A). DNA-supplementation of DNaseI-treated *S. epidermidis* 1457-M10 cultures (Supplementary Fig. 2) had no effect on bacterial uptake as compared to the untreated control (Fig. 5A), providing further evidence that PIA is required for eDNA effectively interfering with phagocytosis. Interestingly, the addition of purified chromosomal DNA isolated from *E. coli* to DNAseI-treated sessile *S. epidermidis* cultures resulted in similar effects compared to chromosomal DNA from *S. epidermidis* (Supplementary Fig. 3).

In addition to the changes in the phagocytic uptake, the removal of eDNA in the biofilms of *S. epidermidis* 1457 also led to strong changes in the hMDM polarization profile, resulting in a significant reduction of CD163^+^, anti-inflammatory macrophages (native control culture: 72.99 ± 4.27% CD163^+^ cells; 1457 DNaseI-treated cultures: 11.45 ± 1.14% CD163^+^ cells) (Fig. 5B), while in parallel the percentage of CD36^+^ hMDM increased compared to the untreated control culture (Supplementary Fig. 4). DNaseI treatment of 1457-M10 and 1457Δ*atlE* cultures had no effect on the proportion of CD163^+^ or CD36^+^ hMDM (Fig. 5B and Supplementary Fig. 4). Consistent with the results of hMDM typing, treatment with DNaseI-strongly improved the ability of *S. epidermidis* 1457 biofilms to induced *IL1B* expression in hMDM, which, though still being lower compared to 1457-M10 infection, was not different from that observed after infection with *S. epidermidis* 1457Δ*atlE* (Fig. 5C). After DNaseI treatment of *S. epidermidis* 1457, though, induction of *IL10* expression was significantly reduced compared to the untreated control, and was indistinguishable from the findings for 1457-M10 or 1457Δ*atlE* mutants (Fig. 5D). DNaseI treatment did not change *IL1B* or *IL10* induction by mutants 1457-M10 and 1457Δ*atlE*.

Addition of exogenous chromosomal bacterial DNA to DNaseI-treated *S. epidermidis* 1457 biofilms restored hMDM anti-inflammatory polarization (61.20 ± 5.00% CD163^+^ cells) (Fig. 5B). In addition, induction of *IL1B* and *IL10* was similar compared to hMDM infected with untreated 1457 (Fig. 5C) (Fig. 5C, D). Interestingly, the addition of exogenous chromosomal DNA to DNaseI-treated *S. epidermidis* 1457Δ*atlE* biofilms was also associated with the induction of an anti-inflammatory hMDM polarization profile (67.61 ± 1.23% CD163^+^ cells; Fig. 5B). In parallel, compared to infection with 1457Δ*atlE* without exogenous DNA-supplementation, *IL1B* was significantly reduced (Fig. 5C), while there was a significant induction of *IL10* expression (mean fold change: 1.156 ± 0.85) expression levels reminiscent of findings in untreated *S. epidermidis* 1457 infection (Fig. 5D). A significant increase of CD163^+^ hMDM and decrease of CD36^+^ hMDM was also noticed after infection with DNaseI-treated DNA-supplemented 1457-M10 sessile cultures (40.37 ± 1.94% CD163^+^ cells), indicating that purified *S. epidermidis* DNA has a general effect on the inflammatory phenotype of hMDM, however, the proportion of CD163^+^ hMDM induced by *S. epidermidis* 1457-M10 supplemented with eDNA was still significantly lower compared to the infection with wild-type 1457 (73,0 ± 4,27% CD163^+^ cells) (Fig. 5B). Compared to infection with untreated control and DNAseI-treated cultures of *S. epidermidis* 1457-M10, exogenously added chromosomal DNA had no significant impact on hMDM *IL1B* or *IL10* expression (Fig. 5C, D).

Taken together, these results indicate that eDNA from the *S. epidermidis* biofilm matrix is responsible for the induction of an antiphagocytic effect and an anti-inflammatory phenotype in hMDM. Although eDNA clearly has biofilm-independent effects on inflammatory macrophage stimulation, failure of exogenously added DNA to protect *S. epidermidis* 1457-M10 from phagocytosis and to induce 1457-like cytokine expression profiles suggests that these responses are dependent on the production of a PIA-containing biofilm matrix scaffold capable of integrating eDNA.

### Toll-like receptor 9-mediated DNA sensing is critical for eDNA-associated macrophage reprogramming

Following up on findings supporting a role for eDNA in failure of macrophages to take up biofilm-forming *S. epidermidis* and induction of an anti-inflammatory response, we hypothesized that eDNA engages dedicated host receptor TLR9 to shape macrophage–*S. epidermidis* interactions^46^.

**Fig. 6:**
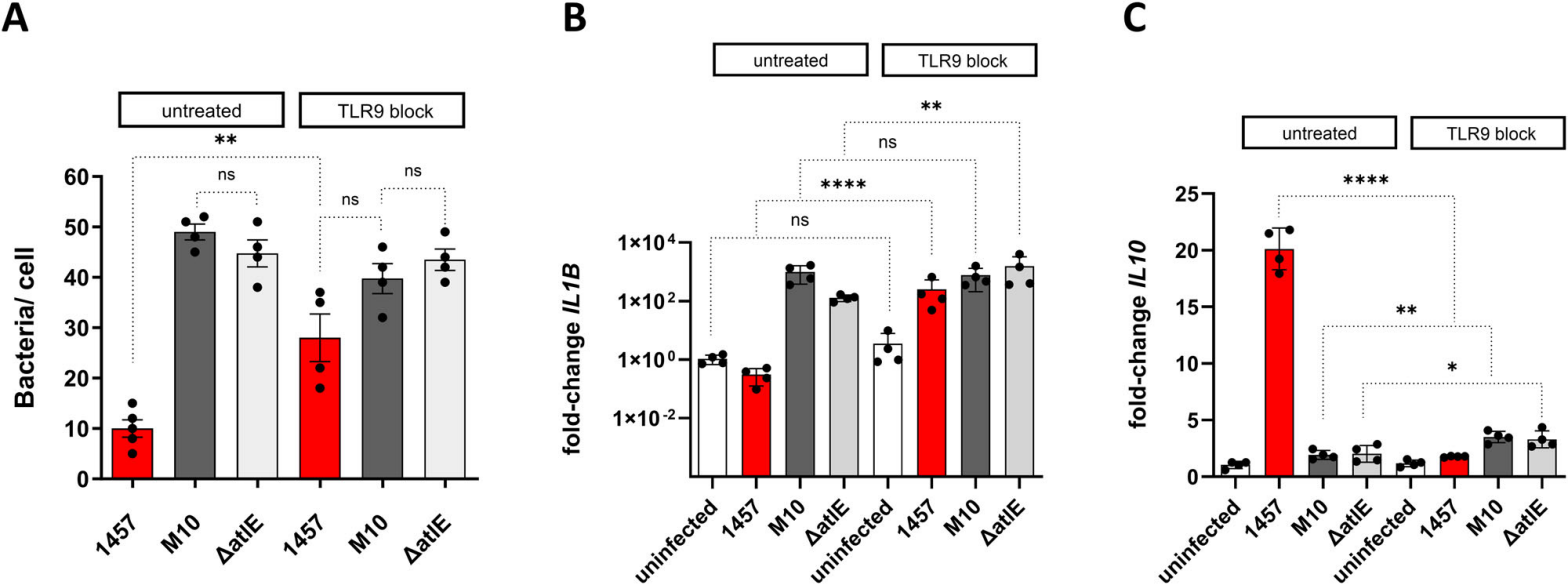
Importance of TLR9 for hMDM activation by *S. epidermidis.* hMDM were treated with TLR9 blocking agent ODN TTAGGG (A151; Invivogen) at 200 nM for 24 h, and subsequently infected with sessile 1457, 1457-M10 and 1457Δ*atlE* cultures for 2 h. **A** Quantification of bacteria per hMDM cell was done using CLSM. Inside-Outside staining was performed by staining total bacteria with DAPI and outside-located bacteria using rabbit α-*Staphylococcus epidermidis* antiserum and a α-rabbit IgG coupled to A568. At least 10 images per experimental condition were analyzed and three individual donors were tested. Columns represent mean number of intracellular bacteria; error bars represent standard deviation. **B**, **C** Quantitative analysis of *IL1B*, and *IL10* expression levels in in ODN TTAGGG treated hMDM after infection with *S. epidermidis* 1457, 1457-M10, and 1457Δ*atlE* using the 2^−ΔΔCt^ method and using *GPDH* as a reference housekeeping gene. Columns represent mean fold changes in expression levels relative to untreated hMDM infected with *S. epidermidis* 1457, and based on the analysis of hMDM from four independent donors. Bars represent standard deviation. Statistical analysis was performed using one-way ANOVA. ns not significant, *P ≤ 0.05; **P ≤ 0.01; ***P ≤ 0.001.

To study the role of TLR9 in *S. epidermidis*– hMDM interactions, a specific inhibitor of TLR9 (ODN TTAGGG (A151), Invivogen) was employed to determine the role of TLR9-mediated DNA sensing for phagocytosis and activation of hMDM after infection with *S. epidermidis*. Compared to the untreated hMDM control, inhibition of TLR9 was associated with a significant, 2.8-fold increase of *S. epidermidis* 1457 uptake (10 ± 3.81 vs 28 ± 9.4 [mean intracellular bacteria/hMDM]) (Fig. 6A). In addition, hMDM infection with *S. epidermidis* 1457 after TLR9 inhibition led to a significantly increased, strong induction of *IL1B* expression compared to the untreated control, being similar compared to *IL1B* expression induced after infection of untreated hMDM with *S. epidermidis* 1457-M10 and 1457Δ*atlE*, respectively (Fig. 6B). Simultaneously, after TLR9 inhibition, *IL10* induction in hMDM infected with *S. epidermidis* 1457 was significantly reduced comparted to untreated control hMDM (Fig. 6C). Blocking of TLR9 did not significantly change phagocytic uptake of *S. epidermidis* 1457-M10 or 1457Δ*atlE* (untreated 1457-M10: 49 ± 3.16, TLR9 inhibition 1457-M10: 39.75 ± 5.90; untreated 1457∆*atlE*: 44.75 ± 5.37, TLR9 inhibition 1457∆*atlE*: 43.5 ± 4.02 [mean bacteria/hMDM]) (Fig. 6A). On the transcriptional level, TLR9 inhibition in hMDM was associated with an increased *IL1B* induction (Fig. 6B). Inhibition of TLR9 resulted in slightly increased *IL10* induction after infection with 1457-M10 and 1457Δ*atlE* (Fig. 6C). Importantly, experiments using ODN TAAGGG control nucleotides found no effect on *S. epidermidis*–hMDM interactions, validating the specificity of the observed, ODN TAAGGG-related effects (Supplementary Fig. 5).

**Fig. 7:**
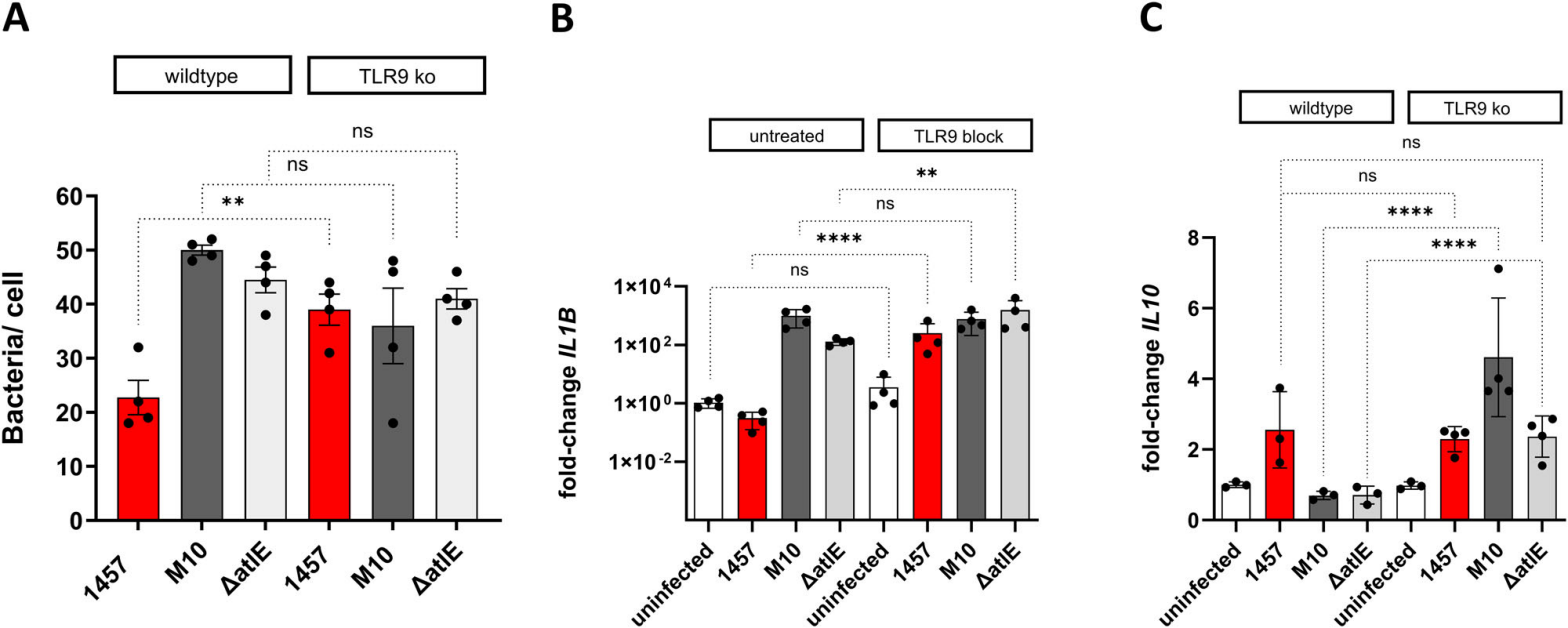
Infection of TLR9 KO cells with sessile *S. epidermidis.* Murine wild-type macrophages NR-9456 and a corresponding TLR9^−/−^ knock-out cell line NR-9569 cell line were infected with 1457, 1457-M10, and 14570Δ*atlE* at an MOI 50 for 2 h. **A** Quantification of bacterial uptake using CLSM. After DAPI staining, and extracellular *S. epidermidis* were specifically detected using a polyclonal rabbit α*-S. epidermidis* antiserum and α-rabbit IgG conjugated to AF488. At least 10 images per strain and cell line were recorded and four individual experiments were performed. Columns represent mean bacteria/cell, error bars indicate standard deviation. Pairwise comparison was done using one-way ANOVA. **B**, **C** Quantitative analysis of *IL1b*, and *IL10* expression in mouse macrophage cell line NR-9456 and TLR9^-/-^ knock-out cell line NR-9569 after infection with *S. epidermidis* 1457, 1457-M10, and 1457Δ*atlE* using *GAPDH* as a reference housekeeping gene. Columns represent mean fold changes in expression levels determined by comparative C(t)-method relative to untreated hMDM infected with *S. epidermidis* 1457, and based on the analysis of hMDM from four independent donors. Bars represent standard deviation. Statistical analysis was performed using one-way ANOVA with Bonferroni correction for multiple testing.ns: not significant, *P ≤ 0.05; **P ≤ 0.01; ***P ≤ 0.001.

Off-target effects are potential confounders in experiments using artificial TLR9 inhibitors. To validate findings obtained by using ODN TAAGGG, *S. epidermidis* infection experiments were carried out using wild-type mouse macrophages NR-9456 and a corresponding TLR9^−/−^ knock-out cell line NR-9569^47^ (Fig. 7). Similar to findings with hMDM, wild-type murine TLR9^+/+^ macrophages NR-9456 engulfed significantly fewer biofilm-forming *S. epidermidis* 1457 (22.75 ± 6.4 [mean number of bacteria/cell]) compared to uptake of mutant *S. epidermidis* 1457-M10 (50 ± 1.8 [mean number of bacteria/cell]) or mutant *S. epidermidis* 1457Δ*atlE* (44.5 ± 4.7 [mean number of bacteria/cell]). Absence of TLR9 in TLR9^−/−^ macrophages was associated with a significant increase of engulfed *S. epidermidis* 1457, rising from 22.7 ± 6.4 to 39 ± 5.7 [mean number of bacteria/cell] (Fig. 7A). There was no difference in bacterial uptake between TLR9^+/+^ and TLR9^−/−^ when infected with 1457-M10 and 1457Δ*atlE* (Fig. 7A).

Analysis of *IL1b* and *IL10* expression revealed that infection of wild-type mouse macrophages NR-9456 with *S. epidermidis* 1457-M10 and 1457Δ*atlE* induced a strong *IL1b* expression, which was slightly lower after infection of TLR9^−/−^ mouse macrophages (Fig. 7B). In contrast, and in line with observations made using ODN TAAGGG to inhibit TLR9 activation in hMDM, infection of murine wild-type macrophages with *S. epidermidis* 1457 did not induce *IL1b* compared to the untreated control (Fig. 7B). In contrast, infection of TLR9^-/-^mouse macrophages NR-9456 with *S. epidermidis* 1457 elicited a significantly induction of *IL1b* expression, which was similar to *IL1b* expression after infection with 1457-M10 and 1457Δ*atlE* (Fig. 7B). Infection of wild-type mouse macrophage NR-9456 with *S. epidermidis* 1457 induced a strong *IL10* expression, while no effects on IL10 induction were observed after infection with *S. epidermidis* 1457-M10 and 1457Δ*atlE* (Fig. 7C). Deletion of *TLR9*, however, did not attenuate *IL10* induction after infection with *S. epidermidis* 1457, while being associated with a significantly increased *IL10* expression after infection with 1457-M10 and 1457Δ*atlE* (Fig. 7C).

Taken together, these findings support the idea that TLR9 transmits eDNA´s anti-inflammatory signals during interactions with eDNA-producing, biofilm-forming *S. epidermidis* 1457. Failure to blunt *IL10* expression in TLR9^-/-^ mouse macrophages indicates potentially important differences between human and mouse macrophages in eDNA-associated activation.

## Discussion

Chronic, subclinical courses with no or only mild overt signs of inflammation are a key clinical feature of device-associated Staphylococcus epidermidis infections. Bacterial persistence despite direct exposure of the invading pathogen to host innate immune defenses, particularly professional phagocytes, is a strong indicator of specific factors governing *S. epidermidis* immune evasion. Indeed, early observations linked the inability of neutrophils and macrophages to eliminate *S. epidermidis* to the production of a biopolymer-containing extracellular matrix and the subsequent assembly of multicellular biofilm consortia^29^. Our study essentially extends the mechanistic understanding of how specific biofilm matrix components interfere with and shape *S. epidermidis*–macrophage interactions. We here show that PIA is necessary but not sufficient for immune evasion, but bacterial eDNA, is an additional necessary component of the biofilm matrix to promote an immune evasive phenotype. Essentially, depending on a functional TLR9, the presence of eDNA results in macrophages’ failure to mount an inflammatory response during interactions with biofilm-forming *S. epidermidis*.

Until now, the inability of macrophages to engulf and clear biofilm-associated staphylococci has largely been attributed to the formation of large aggregates of bacteria, which were supposed to present a physical obstacle to phagocytosis^31,48^. Previous reports have provided evidence that planktonic *S. epidermidis* and *S. aureus* are indeed more efficiently taken up by macrophages compared to bacterial aggregates in biofilm consortia^32,36^, which was further supported by re-sensitizing biofilm-forming *S. epidermidis* to phagocytosis following mechanical disruption of aggregates^36^.

However, the overall importance of frustrated phagocytosis is challenged by results from our comparative analysis of *S. epidermidis* 1457 and its isogenic mutant 1457Δ*atlE*, lacking the major autolysin responsible for eDNA release. In fact, while both strains form surface-adherent biofilms, 1457Δ*atlE* forms even larger aggregates compared to wild-type 1457. Nevertheless, 1457Δ*atlE* is efficiently taken up by hMDM, suggesting that not necessarily biofilm formation or aggregate size alone, but the composition of the biofilm matrix plays a crucial role in shaping interactions between *S. epidermidis* biofilm and macrophages.

Previous work in *S. epidermidis* and other species has suggested that general biofilm biophysical parameters change in relation to the amount of eDNA present in the biofilm matrix^49^, and our comparative analysis of *S. epidermidis* 1457 and 1457Δ*atlE* provides evidence that such differences translate into significant changes in biofilm architecture. Thus, given the existing evidence that physical properties have a profound effect on the phagocytic activity^50,51^, a concept is emerging in which eDNA integration into the biofilm matrix alters the general properties of the bulk bacterial population, resulting in increased resistance to phagocytosis^52^.

eDNA has been identified as an important component of the biofilm matrix in a wide variety of gram-positive and gram-negative microorganisms^53^. Its role in biofilm formation has been studied in great detail for *S. aureus*, supporting an electrostatic net model in which eDNA provides a charged scaffold to support additional biomolecules and ultimately bacterial cells to integrate into multicellular biofilm architectures^54^. Importantly for our model, exogenous addition of chromosomal DNA only restored resistance to phagocytosis in mutant 1457Δ*atlE*, but not in the PIA-negative mutant 1457-M10. The sessile 1457-M10, devoid of any extracellular scaffold, is almost unable to recruit eDNA from solution into the cell population. Consequently, it appears that eDNA effects on phagocytic engulfment are critically dependent on the production of a biofilm matrix that supports eDNA integration and recruitment to the bacterial cell surface. This supports a concept in staphylococci where eDNA not only provides an electrostatic network but is itself dependent on it to ultimately co-organize the biofilm matrix^55^, in turn shaping macrophage interactions. The positive net charge of PIA critically depends on the removal of acetyl moieties, mediated by deacetylase IcaB^56^. It is therefore tempting to speculate that IcaB is also important for PIA-eDNA interactions and the susceptibility of *S. epidermidis* to phagocytosis.

While PIA serves as an eDNA scaffold in *S. epidermidis* 1457, it is important to acknowledge that in *S. epidermidis* isolates employing PIA-independent mechanisms of biofilm accumulation, other matrix macromolecules (e.g., Embp or Aap) may functionally replace PIA^36,57^. This important aspect needs to be studied in full detail using genetic approaches, and by evaluation of the differential importance of intercellular adhesins in collections of clinical *S. epidermidis* isolates.

The detailed analysis of *S. epidermidis*–macrophage interaction based on macrophage typing and gene expression analysis, showed that eDNA present in the biofilm matrix not only affects pathogen uptake, but also profoundly impacts macrophage reprogramming towards a phenotype characterized by the expression of anti-inflammatory *IL10*, and decreased production of pro-inflammatory markers, e.g., L-1β and TNF-α. Induction of anti-inflammatory macrophage phenotypes by biofilm-forming *S. epidermidis* has been reported previously^21,36,38,57,58^. Specifically, using mouse macrophage-like J774A.1 cells in an in vitro biofilm infection model, it became evident that *S. epidermidis* 1457 is a less potent activator of NF-κB and Il-1β production compared to biofilm-negative mutant 1457-M10^36^. Thus, the biofilm growth mode obviously provides additional effectors that ultimately result in anti-inflammatory reprogramming of macrophages when encountering biofilm-forming *S. epidermidis* populations. Several lines of evidence show that Gram-positive and Gram-negative biofilm-forming bacteria per se appear to be capable of inducing macrophages to adopt anti-inflammatory phenotypes^59^. Although clinical information is scarce, evidence from prosthetic joint infections supports the idea that this phenomenon is also clinically relevant in human implant infections^60,61^. Specifically for *S. aureus*, evidence from animal models of biofilm infection show that macrophages in vivo exhibit an anti-inflammatory phenotype^62^, and that production of anti-inflammatory cytokine IL10 critically shapes the immune responses in implant-associated infections^41^. A number of mechanisms have been identified in *S. aureus* that promote anti-inflammatory immune signatures in biofilm-associated implant infections. A key role here was attributed to the changing microenvironment associated with biofilm-forming *S. aureus*, i.e., the development of nutrient gradients, O_2_ consumption and enrichment of metabolites^43,63–65^. E.g., lactate produced by biofilm-forming *S. aureus* induces IL10 production in macrophages via HDAC11 inhibition in vitro and in vivo, causing unchecked HDAC6 activity and increased histone 3 acetylation at the *IL10* promoter^62^. In addition, the *S. aureus* alpha subunit of the ATP synthase AtpA, which is released during cell lysis, is related to control in TNF-α, IL-6 and IL-12p70 production in in macrophages^66^. Finally, the enzyme responsible for c-di-AMP synthesis, DacA, is highly expressed during *S. aureus* biofilm formation, and subsequently released c-DI-AMP facilitates the expression of macrophage type I IFN by a STING-dependent pathway, thereby enhancing anti-inflammatory activity^67^. In this concept where local biofilm matrix environment shapes interactions with professional phagocytes, eDNA might contribute by chelation of calcium ions, an activity that has been documented for *Pseudomonas aeruginosa*^68^. Consecutive sequestration of calcium might then lead to changes in macrophage calcium-sensing receptor activity^69^, in turn modulating pro-inflammatory hMDM activation^70,71^. It appears possible that similar mechanisms are also important in *S. epidermidis* –macrophage interactions. In significant extension to previous work, however, we propose a so far unrecognized mechanism in which eDNA-dependent TLR9 signaling playing a decisive role in reprogramming of hMDM towards an anti-inflammatory phenotype, and the inability to efficiently engulf biofilm-forming *S. epidermidis*.

TLR9 is localized to endosomal compartments and recognizes un-methylated CpG DNA motifs common to bacterial and viral DNA^72,73^. It is likely that during hMDM interactions with sessile *S. epidermidis* and their phagocytic uptake, eDNA bound to the bacterial cell surface can enter the phagolysosomal compartment and activate TLR9. Mutant 1457Δ*atlE* was able to induce an anti-inflammatory hMDM response only after supplementation with eDNA and its co-integration into the biofilm matrix. Mutant 1457-M10, even after the addition of exogenous eDNA, only induced strong inflammatory hMDM responses. Based on these findings, it seems plausible that the absolute amount of eDNA taken up is likely to be critical in determining whether TLR9 activation results in pro-inflammatory or anti-inflammatory hMDM stimulation. Similar to its role in eDNA-associated escape from phagocytosis, PIA would play at least an indirect role in the anti-inflammatory activation of hMDM by providing an essential scaffold for eDNA enrichment. Notably, it cannot be excluded that PIA also plays a direct role, as PIA-induced activation of TLR2^74,75^, may critically modulate TLR9 signaling and downstream signal transduction pathways, ultimately leading to anti-inflammatory priming of hMDM^76^. Moreover, the possible functional importance of additional biomolecules engulfed in their complex with eDNA (e.g., phenol soluble modulins; PSMs^77^) cannot be excluded.

Activation of TLR9 triggers intracellular signaling cascades, ultimately leading to the activation of transcription factors NF-κB and IRFs and subsequent expression of pro-inflammatory cytokines^73,78^. In order to avoid excessive inflammatory activation, however, TLR signaling in general and that of TLR9 in particular is closely controlled in order to balances the net inflammatory activity of macrophages^79,80^. Specifically, the PI3Kγ/AKT/mTor system has been identified to play an important role in shaping inflammatory responses in macrophages^81,82^. In fact, mTOR activation is essential for anti-inflammatory, M2-like macrophage priming through metabolic reprogramming, ultimately leading to IL10 production^83–85^. Thus, it is reasonable to speculate that the mTOR system might play a decisive role for differential macrophage activation patterns depending on the *S. epidermidis* biofilm matrix composition.

Taken together, we provide novel functional insights into the mechanisms underlying *S. epidermidis* protection from macrophage phagocytosis and its ability to blunt pro-inflammatory responses. The importance of eDNA and eDNA-related TLR9 signaling for *S. epidermidis* immune escape fills important gaps in our understanding of how biofilms are able to distort otherwise highly efficient host immune mechanisms. Notably, recent evidence from a *S. aureus* animal model of systemic infection showed that DNase treatment was able to protect against infection through biofilm disruption^86^. Combined with the results of our study, these findings should stimulate research with the potential to translate into clinically relevant new approaches to combat chronic *S. epidermidis* biofilm infections.

## Methods

### Bacterial strains

Experiments were carried out using *S. epidermidis* 1457^87^, a biofilm-forming isolate from a central venous catheter infection, and corresponding isogenic mutants 1457-M10^88^ and 1457Δ*atlE*^89^ (Table 1).

### Biofilm adherence assay

Adherent biofilm formation on plastic (Nunclon, Thermo Fisher Scientific, Waltham, MA, USA) was assessed using the microtiter plate assay as described^13^. In indicated experiments, 500 U DNAseI (Invitrogen, Carlsbad, CA, USA) were added to the culture.

### eDNA quantification

Mature *S. epidermidis* biofilms grown in Petri dishes in 20 ml TSB were detached from the plastic surface. Bacterial suspensions were sonicated at 20% amplitude for 10 s on ice. Bacterial cells were removed by centrifugation, and DNA from the supernatant was isolated by phenol-chloroform extraction. To detect eDNA, RT-q-PCR was carried out using the TaqMan Fast Advanced Master Mix (Thermo Fisher Scientific, Waltham, MA) and primers and probe for *gyrB* (fwd: TGGTCTGCGTTCATTTCACCAAGAC, rev: CTTGCCGATGTTGATGGTGCACA, probe: FAM-GGCGGCTGAGCAATATAAACGTAGCCCGC-BHQ-1) and a LightCycler 480 instrument (Roche Life Science, Basel, Switzerland). The resulting data was analyzed using the Roche LightCycler 480 software package (version 1.5.1.62).

### Semi-quantitative detection of PIA

*S. epidermidis* were grown for 18 h at 37 °C and 180 rpm in 25 ml TSB with appropriate antibiotics. Cultures were sedimented at 5000×*g* for 10 min at 4 °C and washed three times in PBS. The bacteria pellet was re-suspended in 100 µl 1× LDS Sample Buffer (Thermo Fisher Scientific, Waltham, MA, USA), sonified at 20% amplitude for 10 s on ice, and suspensions were incubated at 70 °C for 10 min. 5 µl from serial dilutions were transferred to an activated PVDF membrane (PVDF Transfer Membrane, Thermo Fisher Scientific, Waltham, MA, USA) and incubated overnight at 4 °C in blocking buffer (Protein-Free blocking buffer, Thermo Fisher Scientific, Waltham, MA, USA) in the presence of a polyclonal rabbit α-PIA antiserum at 1:5000 dilution^18,90^. After washing, bound antibodies were detected using an α-rabbit IgG coupled to HRP (Cell Signaling Technology, Danvers, MA) at a 1:10,000 dilution, and made visible using chemiluminescence (ECL Blotting Reagents, Cytiva, Malborough, USA) and the Amersham ImageQuant 800 instrument (Cytivia, Malborough, USA).

### Confocal laser scanning microscopy

Images of macrophages, infected macrophages, and *S. epidermidis* biofilms were acquired using a confocal laser scanning microscope (Leica TCS SP8 X) equipped with a pulsed white light laser, an oil immersion HC PL APO 63× NA 1,4 objective and HyD and PMT detectors, and using the Leica LAS X software. If not indicated otherwise at least 10 Z-stacks per condition were acquired (z distance 250 nm, 2048 × 2048 pixel size) and further analyzed.

### Cell cluster analysis

For determining bacterial cluster sizes *S. epidermidis* strains were grown statically in TSB for 18 h at 37 °C. After vortexing, 10 µl bacteria solution were transferred to staining solution (PBS supplemented with 3% BSA and 300 nM DAPI) in an eight-well chamber slide (ibidi, Graefelfing, Germany) and kept at 4 °C in the dark until imaging. Cluster analysis was carried out as described before^91^.

### Microscopic imaging of adherent biofilms

*S. epidermidis* static cultures were grown in an eight-well chamber slide (ibidi, Graefelfing, Germany) in TSB for 24 h at 37 °C. After careful removal of medium, bacterial samples were fixed in 4% paraformaldehyde (PFA; Electron Microscopy Science, Hatfield, USA) for 10 min and subsequently stained in PBS supplemented with 3% BSA [wt/vol] and 300 nM DAPI for 1 h. Next, samples were embedded in Mowiol and kept in the dark until imaging. Analysis of biofilm biomass, thickness, and surface roughness was performed using Comstat2 plug-in (version Comstat v. 2.1) for ImageJ^92^ and BiofilmQ^93^.

### Immunofluorescence staining of biofilm matrix-associated eDNA

For analysis of spatial eDNA distribution within biofilms bacteria grown on coverslips were carefully washed and fixed in glyoxal solution prepared as described^94^. After blocking unspecific binding sites using BSA at a final concentration of 3% [wt/vol] and incubation for 1 h, samples were incubated with α-dsDNA antibody (Abcam, Cambridge, UK) for 1 h in the presence of BSA (final concentration 1.5% [wt/vol]). After washing, bound antibodies were detected using of α-mouse IgG coupled to A488 (Thermo Fisher Scientific, Waltham, Massachusetts) and 300 nM DAPI (Invitrogen, Carlsbad, CA, USA) for detection of total bacteria. Coverslips were washed three times and mounted on glass slides in Prolong Glass Antifade Mountant (Thermo Fisher Scientific, Waltham, MA).

### Macrophage cell cultures

For the production of primary, human monocyte-derived macrophages (hMDM), peripheral human CD14^+^ blood monocytes were isolated from buffy coats, as described previously^95^. In brief, heparinized whole human blood was added to Lymphocyte Separation Medium (LSM, PAA Laboratories, Cat. No. J15-004) and serum, PBMCs and erythrocytes separated for 30 min at 4 °C and 450×*g*. The milky PBMCs containing interface was isolated, and monocytes were recovered using magnetic α-CD14 beads using appropriate separation columns (Miltenyi Biotec, Bergisch-Gladbach, Germany). Macrophage precursor cells were cultured for at least 6 days in RPMI-1640 (containing 2 mM glutamine and 20% autologous human serum) at 37 °C, 5% CO_2_, and 90% humidity.

Wild-type murine macrophages NR-9456 and a corresponding TLR9 knock-out mutant cell line NR-9569 were obtained from BEI Resources and grown in DMEM modified to contain 10% FBS (Lonza 14-471 F), 2 mM l-glutamine (Invitrogen, Carlsbad, CA, USA), 1 mM sodium pyruvate (Invitrogen, Carlsbad, CA, USA) and 10 µg/mL ciprofloxacin.

### Quantification of bacterial uptake by hMDM

Prior to infection, differentiated hMDM or mouse macrophage cell lines were seeded on coverslips in a total of 5 × 10^5^ cells per well. Native or eDNA-digested coverslips colonized with *S. epidermidis* were added upside down, ensuring direct contact between bacteria and surface-adherent hMDMs. Infection was interrupted by the removal of bacteria-carrying coverslips, and macrophages were immediately fixed using 4% PFA (vol/vol) in PBS for 10 min. Differential detection of intra- and extracellular bacteria was then carried out as described^36^. In brief, extracellular bacteria were detected using a polyclonal rabbit α-*Staphylococcus epidermidis* antiserum at 1:1000 dilution and an α-rabbit IgG coupled to Alexa568 (Thermo Fisher Scientific, Waltham, Massachusetts) at a 1:250 dilution. To specifically stain intracellular bacteria, samples were then permeabilized with 0.1% Triton X-100 (w/v) in PBS for 10 min, and bacteria were stained using the polyclonal rabbit α-*Staphylococcus epidermidis* antiserum (dilution 1:1000) and α-rabbit IgG coupled to Alexa488 (dilution 1:250) (Thermo Fisher Scientific, Waltham, Massachusetts). For visualization of total bacteria, 300 nM DAPI was added. Microscopic analysis of intra- and extracellular bacteria was assessed after mounting the samples in Prolong Glass Antifade Mountant (Thermo Fisher Scientific, Waltham, MA, USA).

### Image stream

Infected macrophages were harvested at indicated time points, treated for 20 min with Fc blocking reagent (BD Biosciences, Heidelberg, Germany; diluted 1:50 in FACS buffer), and stained using α-CD14 AF512 (antibodies-online.com). Samples were transferred to tubes and directly analyzed using the Image Stream Flow Cytometer (Amnis ImageStreamX Mk II, Cytek, Fremont, CA). Data were analyzed using the Image Stream software package.

### Analysis of hMDM differentiation using flow cytometry

To characterize pro- and anti-inflammatory hMDM differentiation, macrophages were stained using mouse α-iNOS (Abcam, Cambridge, UK), mouse α-MHCII (BioRad, Hercules, USA), α-CD14 AF512 (antibodies-online.com, Aachen, Germany), mouse α-CD36 AF568 (antibodies-online.com, Aachen, Germany), mouse α-CD68 (antibodies-online.com, Aachen, Germany), mouse α-CD86 (BioLegend, San Diego, USA), mouse α-150 (abcam, Cambridge, UK), mouse α-CD163 AF647 (antibodies-online.com, Aachen, Germany) IgG diluted 1:50 in FACS buffer (0,1% BSA and in PBS). In addition, live/dead staining was carried out (Invitrogen, Carlsbad, CA, USA). Samples were examined using the FACSAria Fusion instrument (BD Biosciences, Heidelberg, Germany). Data were analyzed with FlowJo v10.10.0 software package.

### Interference with TLR activation

To specifically inhibit TLR9, ODN TTAGGG (A151) at a concentration of 100 µM, and the corresponding negative control (Invivogen, Toulouse, France) were employed. hMDM were treated with these reagents 24 h prior to infection.

### qPCR gene expression analysis

To quantify expression of *IL1B*, *TNFA*, *IL10*, RNA was extracted from macrophage cultures using the RNeasy Mini Kit (Qiagen) following the manufacturer’s instructions. RNA was quantified with a Qubit fluorometer (Thermo Fisher Scientific, Waltham, Massachusetts) followed by digestion of residual DNA with a DNA-Free Kit (Invitrogen, Carlsbad, CA, USA). Reverse transcription of 1 µg total RNA was carried out using the iScript cDNA Synthesis Kit (BioRad, Hercules, USA), followed by RT-q-PCR reaction using the TaqMan Fast Advanced Master Mix (Thermo Fisher Scientific, Waltham, MA) and specific primers. For the determination of *IL1B*, *TNF*A and *IL10* levels the TaqMan Gene Expression Assay for human *IL1B* (Hs00174097_m1), *TNFA* (Hs01113624_g1) and *IL10* (Hs00961622_m1) were used. As a reference, the TaqMan Gene Expression Assay for GAPDH (Hs02758991_g1) was applied (all assays purchased from Thermo Fisher Scientific). PCR was performed in the LightCycler 480 (Roche Life Science), and the resulting data was analyzed using the enclosed software (Roche LightCycler 480 software; Software release 1.5.1.62). For relative quantification of gene expression, ΔCt and 2^-ΔΔCt^ method was employed^96^.

### Ethics statement

Approval for the analysis of anonymized blood donations (WF-015/12) was obtained by the Ethical Committee of the Ärztekammer Hamburg (Germany).

## Supplementary information

Supplementary information
