# Supplementary material for: Staphylococcus epidermidis alters macrophage polarization and phagocytic uptake by extracellular DNA release in vitro
Source: NPJ Biofilms Microbiomes. 2024 Nov 20;10:131. doi: 10.1038/s41522-024-00604-7 (PMC11579364; doi:10.1038/s41522-024-00604-7)
Supplement: Supplementary file 1 — Supplementary information [file 41522_2024_604_MOESM1_ESM.pdf]

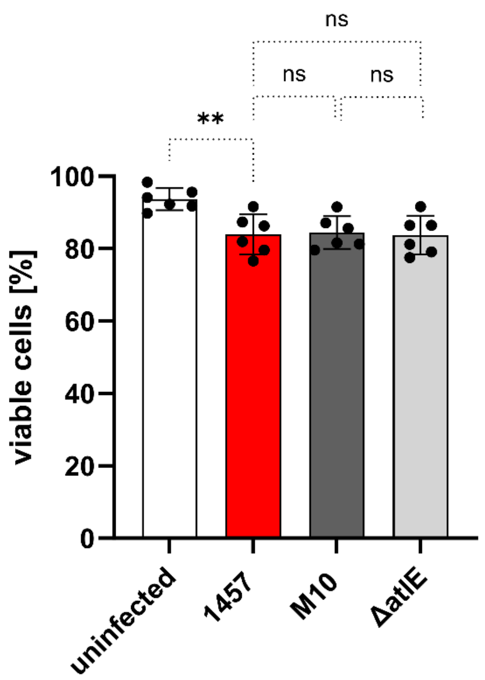

Supplementary Figure 1: hMDM viability after infection with *S. epidermidis*. Macrophages were infected for 2 h with sessile 1457, 1457-M10 and 1457Δ*atlE* cultures. hMDM viability was assessed using SYTOX Green Nucleic Acid Stain according to manufacturer's instructions. Bars represent mean proportion of viable hMDM from three independent experiments, error bars indicate standard deviation. Pairwise comparison was done using one-way ANOVA. ns: not significant, *$p \leq 0.05$; **$p \leq 0.01$; *** $p \leq 0.001$

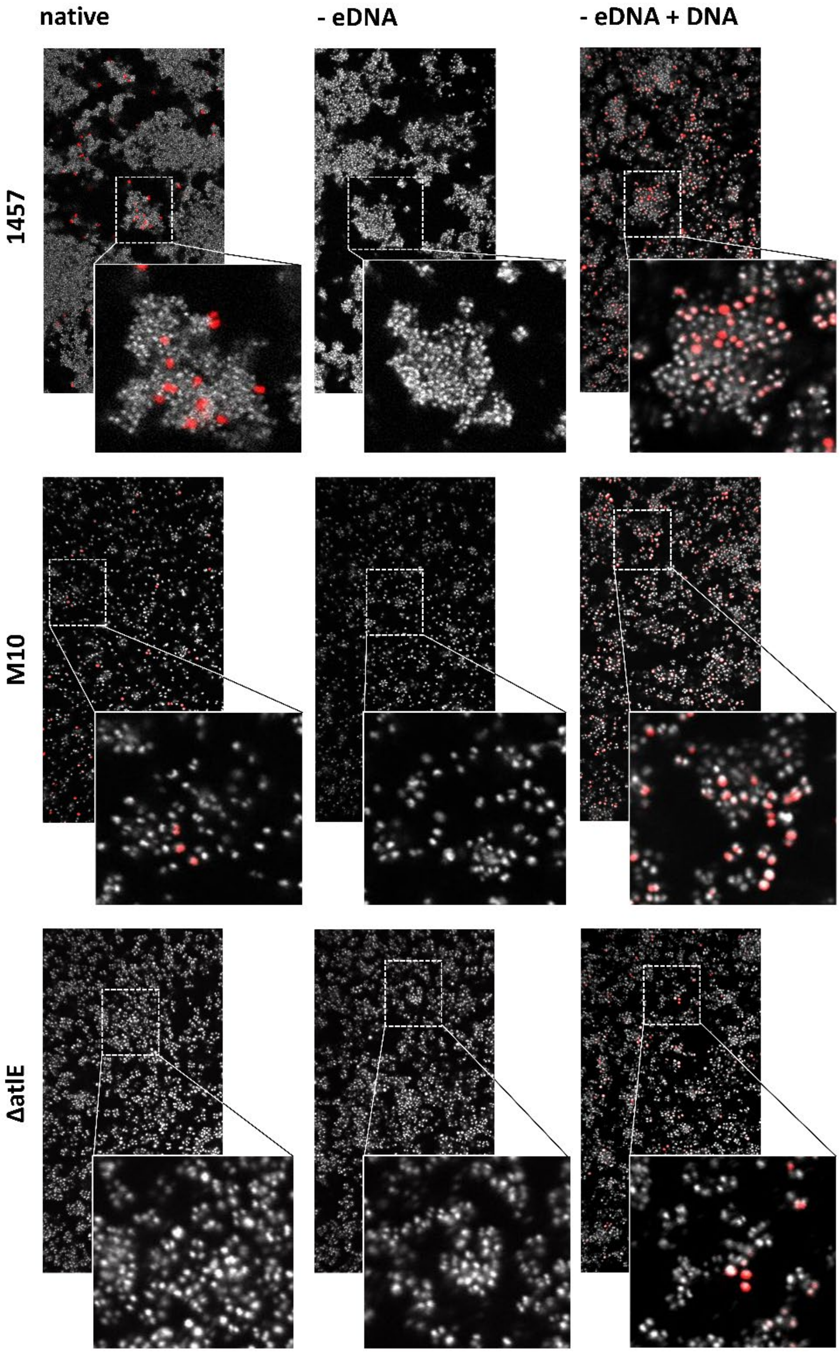

Supplementary Figure 2: DNAseI treatment of native *S. epidermidis* cultures and eDNA supplementation. *S. epidermidis* strains 1457, 1457-M10 and 1457Δ*atlE* were grown statically overnight. eDNA was removed using DNAseI. Chromosomal *S. epidermidis* DNA was added at a concentration of 1000 µg/ml. Bacteria were detected using DAPI. Extracellular DNA was stained using a mouse α-dsDNA IgG antibody and α-mouse IgG coupled to AF568. Images were acquired using CLSM. Representative images of three biological replicates are shown. Scale bar: 5 µm.

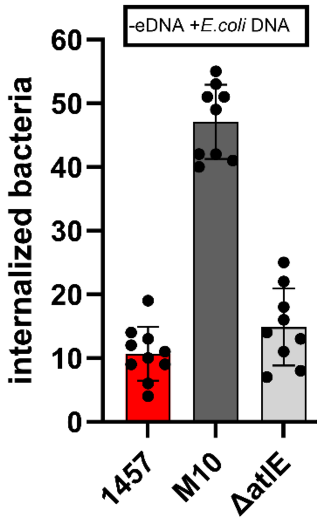

Supplementary Figure 3: Uptake of *S. epidermidis* supplemented with exogenous chromosomal *E. coli* DNA into hMDM. Sessile *S. epidermidis* cultures were first treated with DNAseI to remove endogenous eDNA, and subsequently purified chromosomal *E. coli* DNA was added at a concentration of 1000 µg/mL. Bacterial uptake was assessed using CLSM. At least 10 images per experimental condition were analyzed and three individual donors were tested. Columns represent mean number of engulfed bacteria, error bars indicate standard deviation. Pairwise comparison was done using one-way ANOVA. ns: not significant, *$p \leq 0.05$; **$p \leq 0.01$; *** $p \leq 0.001$.

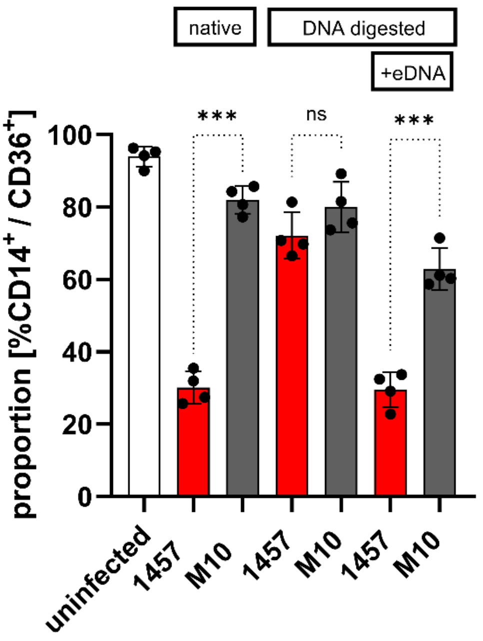

Supplementary Figure 4: Polarization of hMDM after eDNA removal of biofilm. After 2 hours infection with native, DNase-treated and DNase-treated + DNA supplemented 1457 and 1457-M10 sessile cultures pro-inflammatory polarization was checked by staining cell surface markers with α-CD14 FITC and α-CD36 PE antibodies. FACS analysis was performed of four independent donors. Bars represent mean ± standard deviation. Pairwise comparison was done using one-way ANOVA. ns: not significant, *$p \leq 0.05$; **$p \leq 0.01$; *** $p \leq 0.001$

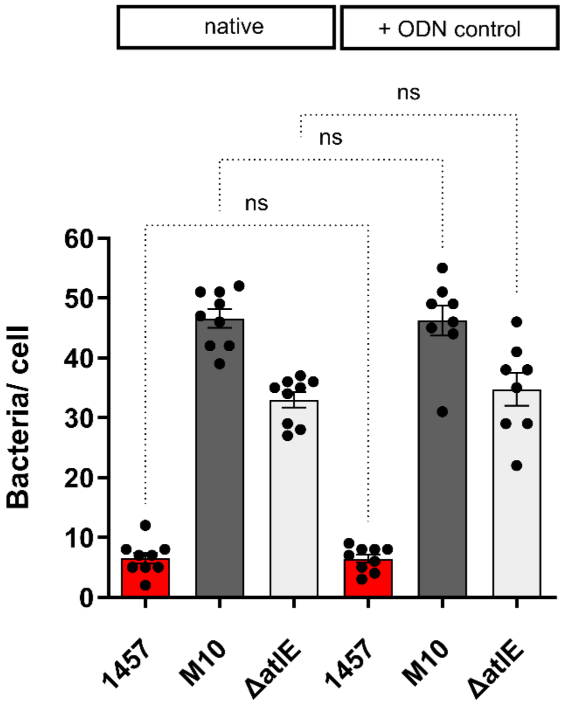

Supplementary Figure 5: Effect of ODN TTAGGG negative control nucleotide on *S. epidermidis* phagocytosis. hMDM were treated with a 15mer ODN TTAGGG control nucleotide (Invivogen) at a concentration of 200 nM for 24 h, and subsequently infected with sessile sessile 1457, 1457-M10 and 1457Δ*atlE* cultures for 2 hours. Quantification of bacteria per hMDM cell was done using CLSM. Inside-Outside staining was performed by staining total bacteria with DAPI and outside located bacteria using rabbit α-*Staphylococcus epidermidis* antiserum and a α-rabbit IgG coupled to A568. At least 10 images per experimental condition were analyzed and three individual donors were tested. Columns represent mean number of intracellular bacteria, error bars represent standard deviation. Pairwise comparison was done using one-way ANOVA. ns: not significant, *$p \leq 0.05$; **$p \leq 0.01$; *** $p \leq 0.001$
